# Effects of diagnostic ultrasound with cRGD-microbubbles on simultaneous detection and treatment of atherosclerotic plaque in ApoE^−/−^ mice

**DOI:** 10.3389/fcvm.2022.946557

**Published:** 2022-07-22

**Authors:** Shengcun Guo, Shengye Zhang, Kui Chen, Xi Chen, Fudong Hu

**Affiliations:** Department of Cardiology, The First Affiliated Hospital of Zhengzhou University, Zhengzhou, China

**Keywords:** atherosclerotic plaque, diagnostic ultrasound, microbubbles, activated platelets, GP IIb/IIIa receptor

## Abstract

**Background:**

Atherosclerotic vulnerable plaque is the leading cause of acute fatal cardiovascular events. Thus, early rapid identification and appropriate treatment of atherosclerotic plaque maybe can prevent fatal cardiovascular events. However, few non–invasive molecular imaging techniques are currently available for the simultaneous detection and targeted treatment of atherosclerotic plaques. We hypothesized that diagnostic ultrasound (DU) combined with cyclic Arg-Gly-Asp-modified microbubbles (MB_R_) could provide targeted imaging and dissolution of activated platelets to identify advanced atherosclerotic plaques and improve plaque instability.

**Methods:**

Three mouse models, apolipoprotein E-deficient mice on a hypercholesterolemic diet (HCD) or normal chow diet and wild-type mice on an HCD were used. The most appropriate ultrasonic mechanical index (MI) was determined based on the expression of GP IIb/IIIa in sham, DU alone and DUMB_R_-treated groups at MI values of 0.5, 1.5, and 1.9. The video intensity (VI) values, activated platelets and plaque instability were analyzed by ultrasound molecular imaging, scanning electron microscopy and histopathological methods.

**Results:**

We found that the VI values of ultrasound molecular imaging of MB_R_ were positively correlated with plaque GP IIb/IIIa expression, vulnerability index and necrotic center / fiber cap ratio. 24 h after treatment at different MIs, compared with those of the other groups, both the VI values and GP IIb/IIIa expression were significantly reduced in MI 1.5 and MI 1.9 DUMB_R_-treated groups. The plaque vulnerability index and necrotic center / fiber cap ratio were significantly decreased in MI 1.5-treated group, which may be due to targeted dissolution of activated platelets, with a reduction in von Willebrand factor expression.

**Conclusion:**

DUMB_R_ targeting GP IIb/IIIa receptors could rapidly detect advanced atherosclerotic plaques and simultaneously give targeted therapy by dissolving activated and aggregated platelets. This technology may represent a novel approach for the simultaneous identification and treatment of atherosclerotic plaques.

## Introduction

Cardiovascular disease is the leading cause of morbidity and mortality worldwide (1). The typical underlying pathology is the sudden rupture or erosion of an atherosclerotic vulnerable plaque, followed by platelet activation, aggregation and subsequent thrombus formation (2). Accumulating evidence suggests that activated platelets within plaques are significantly correlated with plaque vulnerability (3, 4). Therefore, the rapid identification and immediate therapy of activated and aggregated platelets may be critical for the diagnosis and treatment of atherosclerotic plaques. Currently, activated platelets can be detected by imaging methods, but approaches applied to stabilize vulnerable plaques are limited to the application of antiplatelet drugs, such as aspirin, clopidogrel, ticagrelor, GP IIb/IIIa receptor inhibitors and plasminogen activators, in the clinic. These agents cannot reduce adverse cardiovascular events and may have drug-associated adverse effects after either systemic or oral administration, leading to drug resistance in the long term (5–7). Therefore, it is vital to develop a non–invasive imaging approach that allows the simultaneous diagnosis and treatment of activated platelets, as well as tracking of the therapeutic effect.

To date, a non–invasive molecular imaging method that is capable of synchronous detection and targeted therapy of high-risk plaques is not available. Among all imaging modalities, ultrasound molecular imaging, also known as diagnostic ultrasound (DU) combined with microbubbles (MB), has the advantages of being non–invasive, free of radiation-associated risk, available, inexpensive, and inherently real-time, prompting us to use it for the development of a specific theranostic approach (8). Recently, our previous study successfully used DU + MB to target activated platelets to identify high-risk plaques (4); however, whether the DU + MB technology could be able to simultaneously detect and treat atherosclerotic plaques by targeting activated and aggregated platelets in chronic atherosclerotic plaques remains unknown. Multiple studies demonstrated that DU + MB treatment can significantly dissolve acute intravascular thrombi in large vessels and microthrombi (platelet-rich) in microvasculature in models of acute myocardial infarction (9–12), and whether DU in combination with MB treatment could be applied in the chronic atherosclerosis remains unclear.

The glycoprotein (GP) IIb/IIIa complex, known as αIIbβ3 integrin, is the major receptor expressed on the surface of activated platelets, and mediates platelet bridging, subsequent aggregation and thrombus formation *via* a high-affinity state (13, 14). Our previous studies demonstrated that the GP IIb/IIIa receptor of activated platelets is significantly correlated with plaque vulnerability (4). These features render the GP IIb/IIIa receptor an ideal target for both molecular imaging of aggregated platelets and targeted therapy of atherosclerotic plaques. We developed a cyclic Arg-Gly-Asp (RGD)-modified MB (MB_R_) with 30-fold higher affinity and stability in binding the GP IIb/IIIa complex than linear MB and used MB_R_ for the targeted detection of activated platelets and thrombi in large arteries (4, 15), without facilitating platelet activation and crosslinking, which might cause thrombosis (16). These targeted MB_R_ bind GP IIb/IIIa on activated platelets, allowing real-time imaging and safe dissolution of aggregated platelets, thereby stabilizing atherosclerotic plaques.

The current study examined whether DU combined with MB_R_ (DUMB_R_) is capable of simultaneous targeted imaging and dissolution of aggregated platelets to detect advanced atherosclerotic plaques and improve plaque instability.

## Methods

### Animal groups

The animal study protocols were approved by the Institutional Animal Care and Use Committee of Zhengzhou University. The present study conformed to the rules of the Guide for the Care and Use of Laboratory Animals published by the U.S. National Institutes of Health (NIH 8th edition, 2011) and the guidelines for experimental atherosclerosis studies described in the American Heart Association (AHA) Statement.

Male wild-type C57BL/6 and apolipoprotein E-deficient (ApoE^−/−^) mice were purchased from Vital River Laboratory Animal Technology Co. Ltd. (Beijing, China). The following groups were used: ApoE^−/−^ mice fed a hypercholesterolemic diet (ApoE^−/−^ + HCD, *n* = 180), ApoE^−/−^ mice fed a normal laboratory diet (ApoE^−/−^ + NLD, *n* = 12), and wild-type C57BL/6 mice fed an HCD (C57BL/6 + HCD; *n* = 12). For experiments, 6-week-old mice were had free access to their respective diets (purchased from Medicience Biomedical Co. Ltd.) and tap water for up to 30 weeks.

### Experimental protocol

To observe the correlation between GP IIb/IIIa and plaque instability, 6 mice per group were randomly selected for the contrast enhanced ultrasound tested experiments and another 6 mice for blockade experiments. The abdominal aorta was collected, and hematoxylin and eosin (H & E) and immunohistochemical staining were performed.

To establish an appropriate DU mechanical index (MI), 36 HCD-fed ApoE^−/−^ mice were randomly divided into a sham group and five treatment groups (*n* = 6 per group). The sham group was subjected to the DU transducer in the “off” setting and injected with MB_R_. The five treated groups were divided into three DUMB_R_-treated groups, a DU-only-treated group and a DU+negative control microbubble (DUMB_C_) treatment group. The DUMB_R_-treated groups were insonated with different MI values (0.5, 1.5, and 1.9) and injected with MB_R_, while the DU-only-treated group was insonated with an MI of 1.9 in the absence of any microbubbles, and DUMB_C_-treated group was insonated with an MI of 1.9 and received an injection of MB_C_. The mice were subjected to targeted ultrasound molecular imaging before and 24 h after the treatment. Then, the mice were sacrificed 24 h after treatment and the abdominal aortas were collected for immunohistochemistry. In addition, another 12 HCD-fed ApoE^−/−^ mice were randomly divided into DUMB_R_-treated groups with an MI of 1.5 or 1.9. At 24 h after treatment, the abdominal aortas were collected for scanning electron microscopy.

To evaluate the influence of DUMB_R_ treatment on surrounding normal tissue surrounding the abdominal aorta, samples of the abdominal skin and mesentery from the different groups were obtained for H & E staining. In addition, to assess the bleeding time of DUMB_R_, 5 mm of the mouse tail was cut off from the tip and immersed in saline at 37°C. The bleeding time was determined by the time required to stop visible blood flow for at least 1 min.

To explore the mechanism by which plaque instability was reversed, 72 HCD-fed ApoE^−/−^ mice were randomly selected to receive the DUMB_R_ treatment with an appropriate MI that was previously determined. The abdominal aortas were collected before and 0 h, 24 h and 8 weeks after DUMB_R_ treatment for H&E, immunohistochemistry, histoimmunofluorescence and scanning electron microscopy.

**Figure 1:**
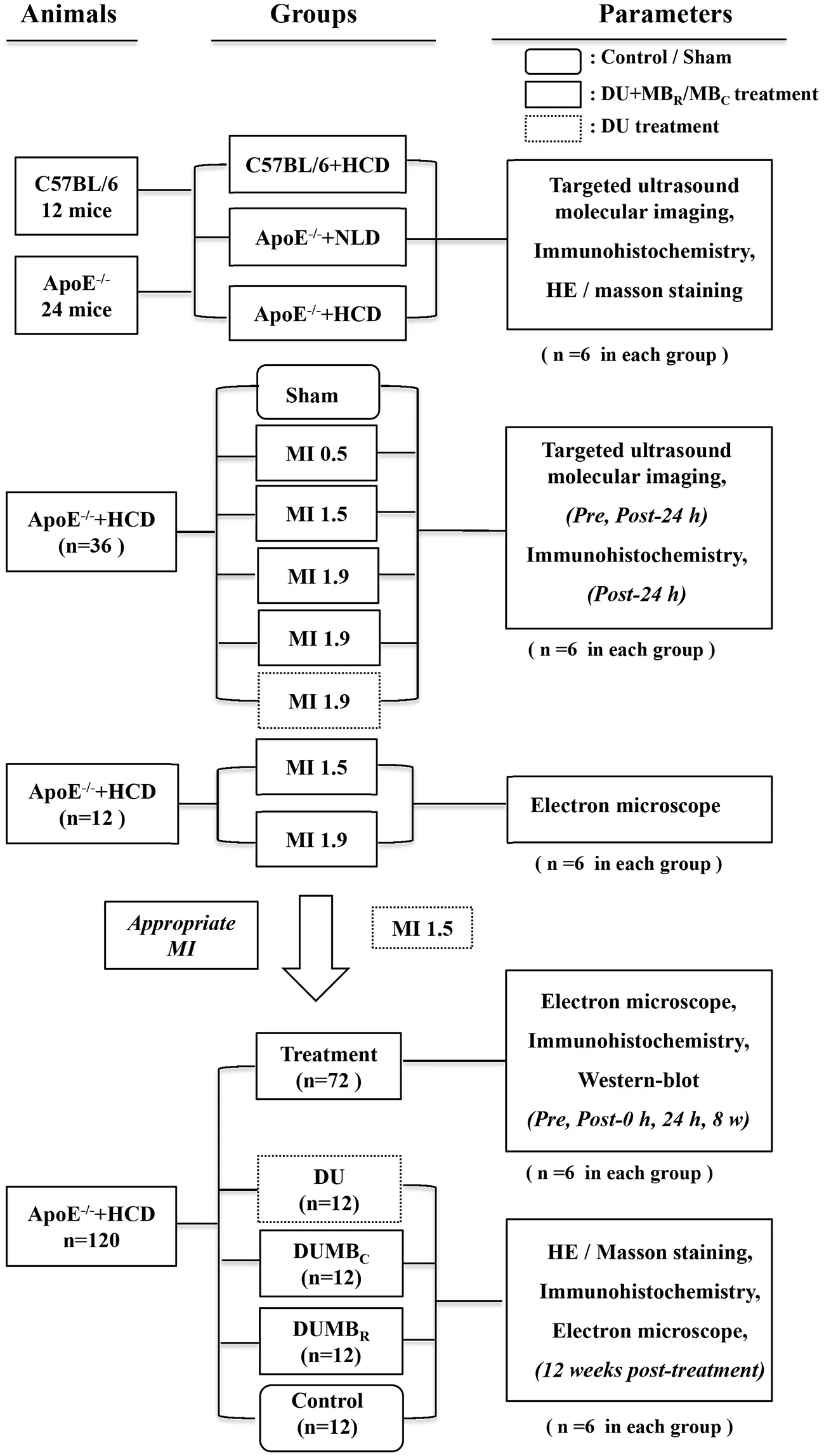
Illustration of the experimental protocol.

To evaluate the therapeutic effect of DUMB_R_ treatment on plaques, 48 HCD-fed ApoE^−/−^ mice were randomly divided into DUMB_R_ treatment (*n* = 12), DUMB_C_ treatment (*n* = 12), DU only (*n* = 12) and control (*n* = 12) groups; the mice were continued to be fed a laboratory diet for 12 weeks after receiving the respective interventions. Plaque instability was assessed by H & E/Masson's trichrome staining and IHC at 12 weeks after treatment. The number of activated platelets aggregated on abdominal aortic plaque was counted by scanning electron microscopy. The animal grouping and experimental protocol are illustrated in Figure 1.

### Microbubble preparation and characterization

The generation of GP IIb/IIIa-targeted and negative control MBs (MB_R_ and MB_C_, respectively) and validation of the binding characteristics were performed as previously described (15). The concentration, and particle size distribution of the two types of MBs were characterized with a Coulter counter (Beckman Coulter Inc., Brea, CA, United States).

### Ultrasound molecular imaging of atherosclerotic plaques

The mice underwent ultrasound molecular imaging performed with a Sequoia ultrasound system using a linear-array probe (15L7; MI: 0–1.9; frequency: 7–15 MHz; Siemens Medical Systems) with Cadence contrast pulse sequencing as previously described (4). Briefly, imaging was performed at a centerline frequency of 7.0 MHz and MI of 0.18 in the longitudinal axis view. Real-time images were obtained for 6 min after an intravenous injection with a bolus of microbubbles. Then, the MI was transiently increased to 1.0 for 3 s to destroy adhered MBs, and subsequent post-destruction images were acquired at an index of 0.18 to obtain the background images of residual bubbles present in the bloodstream. The averaged post-destruction contrast frames were digitally subtracted from the averaged pre-destruction frames at 6 min to identify the signal attributable to retained microbubbles using Yabko MCE2.7 image analysis software (University of Virginia, Charlottesville, VA, United States) and then color-coded; the background-subtracted video intensity was measured from the abdominal aorta (4, 17). To confirm the correlation of video intensity value of UMI with plaque GP IIb/IIIa or vulnerability, contrast enhanced ultrasound was performed in C57BL/6 + HCD, ApoE^−/−^ + NLD and ApoE^−/−^ + HCD mice (*n* = 6 each), with an intravenous injection of 1 × 10^6^ MB_R_ or negative control MB (MB_C_) in a total volume of 0.1 ml in a random order. To demonstrate the high affinity of MB_R_ for the GP IIb/IIIa receptor *in vivo*, a competitive GP IIb/IIIa antagonist, Eptifibatide (integrilin), was injected at a dose of 1.8 μg/g to saturate integrins, followed by an injection of MB_R_ or MB_C_ and contrast enhanced ultrasound. To determine the appropriate MI, mice from the sham or DUMB/DU groups were observed by contrast enhanced ultrasound before treatment and continued feeding until 24 h after treatment; contrast enhanced ultrasound was performed again with an injection of a bolus of 1 × 10^6^ MB_R_ as described above (4, 18).

### DUMB_R_ treatment of atherosclerotic plaques

A Siemens Acuson Sequoia with a 15L7 linear-array transducer (frequency range, 7–15 MHz; elevation plane, 5 mm) and contrast pulse sequencing (Siemens Medical Systems) was employed as the ultrasonic therapy device. The mice in the sham group were not subjected to the DU treatment, while the mice in the DUMB_R_, DU only and DUMB_C_ groups were treated with DU. The transducer was fixed on a steel stand with a scale and held 0.5 cm over the abdominal skin with ultrasound gel. In the DUMB_R_/DUMB_C_-treated groups, during the infusion of microbubbles (0.008 mL/min) *via* a tail vein catheter, contrast pulse sequencing was utilized to visualize the abdominal aortic longitudinal axis at an MI of 0.18 until evidence of contrast filling with the whole abdominal aorta was observed. The MI was increased to high MIs (0.5, 1.5, and 1.9) for 30 s while scanning the whole abdominal aorta. Then, the MI was decreased to 0.18, and real-time imaging was repeated until the evidence as above was observed again. High-MI impulses were repeated for 30 s. This sequence of high- and low-MI imaging was repeated for 30 min (10, 19). The sound beam was aimed to expose the whole abdominal aorta by manually swinging the probe.

### Detection of activated platelets by scanning electron microscopy

Abdominal aorta tissue samples were fixed with 2.5% glutaraldehyde and prepared for scanning electron microscopy following a standard procedure. Activated platelets adhered and aggregated on the surface of the vessel lumen were observed by SEM (S-3000N; Hitachi, Tokyo, Japan) at 20 kV. The number of platelets was counted per field (25.2 × 25.2 mm) in 10 optical fields.

### Fluorescence staining

Histoimmunofluorescence was performed as described previously (20). Briefly, 3 μm sections were permeabilized, blocked and then incubated with rabbit polyclonal antibody against mouse CD31 (Abcam, Cambridge, MA, United States) to detect endotheliocytes. An Alexa Fluor 488-conjugated secondary antibody (Invitrogen) was used. Cell nuclei were counterstained with DAPI. Sections were imaged using a fluorescence microscope (Olympus, Tokyo, Japan). The number of angiogenesis was calculated in 10 randomly selected 400 × high-power fields from 6 separate sections per sample.

### Histology and immunohistochemistry

The samples of the abdominal aorta used for the histopathologic examination were fixed in 4% paraformaldehyde, dehydrated, paraffin-embedded, and sectioned at 3 μm. Five representative serial sections were selected in each site in the proximal, intermediate, and distal ends of the abdominal aortas of each animal. H&E staining, Masson's trichrome staining and immunostaining were performed as previously described (4). Images were observed under an optical microscope.

The fiber lipid deposition and collagen fiber were assessed by H&E and Masson's trichrome staining (MST-8003; Matxin Labs Pvt., Ltd., Bangalore, India). For immunohistochemistry staining, the primary antibodies included rabbit anti-mouse polyclonal antibodies specific for α-smooth muscle actin (α-SMA) (ab5694), CD68 (ab125212), GP IIb integrin (ab63983), von Willebrand factor (vWF) (ab9378), tissue factor (TF) (ab104513), monocyte chemotactic protein (MCP-1) (ab25124) and vascular cell adhesion molecule-1 (VCAM-1) (monoclonal antibody; ab134047, all from Abcam), and a rat anti-mouse monoclonal antibody specific for TER-119 (550565, BD Biosciences). The secondary antibodies were peroxidase-conjugated anti-rabbit or rat IgG. Incubation without primary antibodies and/or with isotype-matched immunoglobulins was used as a negative control for all immunostaining. To determine the specificity of platelet staining, the primary antibody was substituted with the GP IIb/IIIa antigen-antibody complex as a pre–absorption negative control.

### Plaque quantification of histopathologic indicators

The necrotic center/fiber cap (NC/FC) ratio was calculated by the area of lipid deposition and collagen fiber which were measured by planimetry. The areas of positive staining for SMCs and macrophages were quantified as percentages of positive staining within the total plaque area using Image-Pro Plus (IPP, Media Cybernetics, Rockville, MD, United States). The plaque vulnerability index was calculated using the following formula: vulnerability index = (macrophages % + lipids %) / (SMCs % + collagen %) (4, 21). The content of GP IIb/IIIa was measured by the following two methods as previously described: the percentage of GP IIb/IIIa expression in the plaque and the percentage of GP IIb/IIIa coverage in the endothelium (4). The extravasation of erythrocytes in plaques was quantified by counting the average number of erythrocytes in 10 randomly selected 400 × high-power fields from 6 separate sections per sample. The vWF content in plaques was measured by counting the average number of vWF-positive endothelial cells. In addition, the areas of positive immunoreactivity for other factors, such as TF, VCAM-1 and MCP-1, in plaques were quantified as described above for SMCs.

The histopathologic indicators were quantified at the three selected sites and averaged per section per site. All measurements and analyses of the ultrasound molecular imaging, scanning electron microscopy, histology, and immunolabeling data were performed by two individuals who were blinded to the experimental design.

### Western blot analysis

Proteins were obtained from whole abdominal aorta homogenates. Immunoblotting was performed to assess the expression of TF, VCAM-1 and MCP-1. The immunoreactive bands were visualized by Odyssey Software (version 1.2; LI-COR, Lincoln, NE, United States), and quantified using Image J (NIH, Bethesda, MD, United States) as previously described (22).

### Statistical analysis

Data were analyzed using SPSS v.13.0 (SPSS, Inc., Chicago, IL, United States) and are presented as the mean ± standard deviation. The normal distributions of continuous variables were confirmed by the Kolmogorov-Smirnov test. For the variables with normal distributions, 2-tailed Student's *t*-test (for comparisons between two groups) or one-way analysis of variance (for comparisons among three or more groups or time points), followed by Bonferroni (for comparisons with equal variance) or Dunnett's T3 (for comparisons without equal variance) *post-hoc* tests were used for the statistical analysis. For the variables with non–normal distributions, the Mann-Whitney rank sum test was used. Spearman's rank correlation was used to assess the linear correlation between selected variables. A *P* value<0.05 was considered statistically significant.

## Results

### MB characterization

The two types of MBs used in this study had a mean diameter of approximately 2.55 μm, and the mean concentrations were approximately 1.15 × 10^9^ MB per ml. Supplementary Table 1 summarizes the parameters of MB_C_ and MB_R_. There were no significant differences in the mean diameter or concentration between the two types of MB (*P* > 0.05). The particle size distribution of MB_C_ and MB_R_ was shown by Supplementary Figures 3A,B.

### The correlation among GP IIb/IIIa expression, plaque vulnerability and video intensity of ultrasound molecular imaging

**Figure 2:**
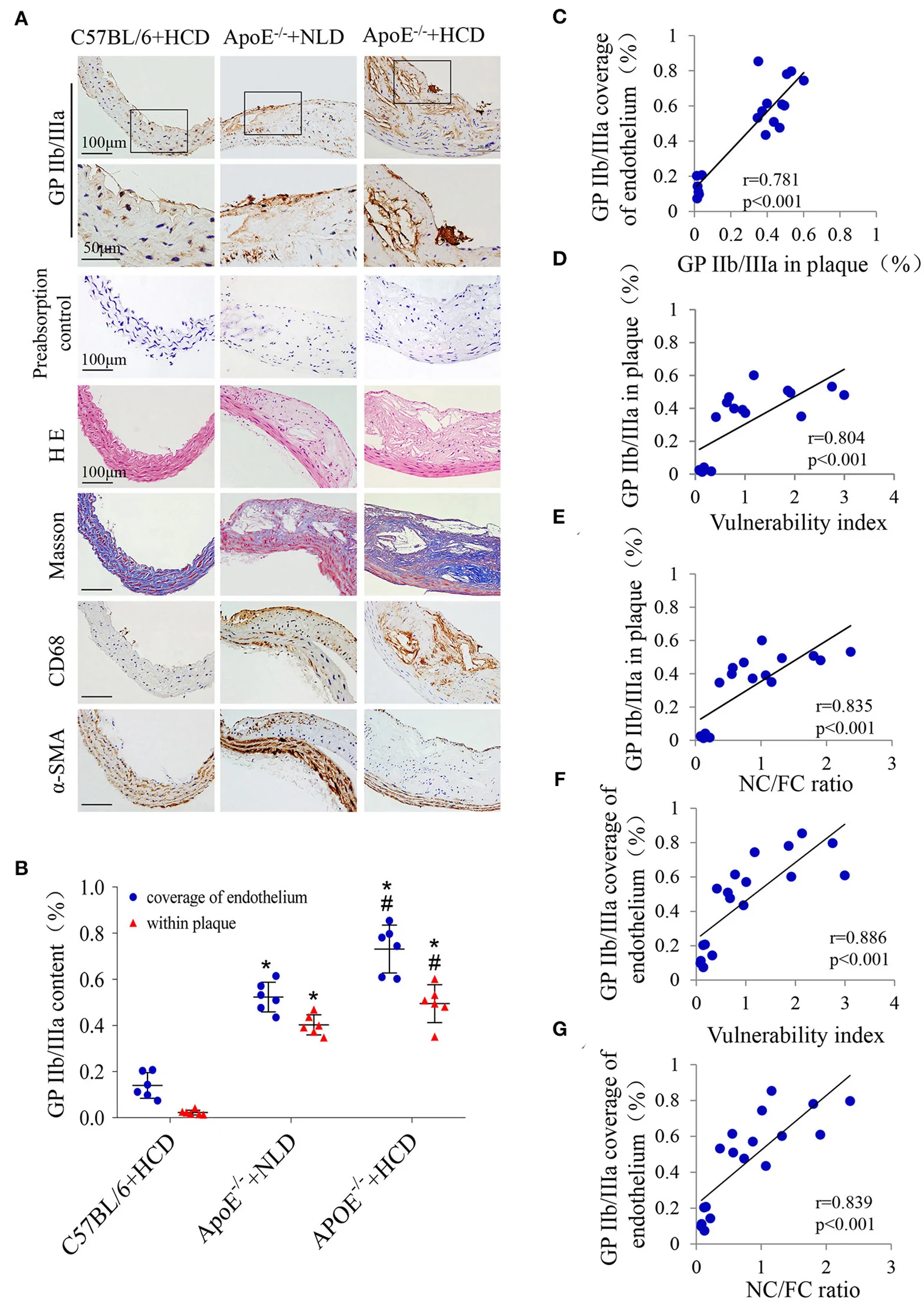
Correlations between GP IIb/IIIa expression and the plaque vulnerability indicators. **(A)** Representative images of aortic tissue visualized by H & E and Masson's trichrome staining or labeling with antibodies against GP IIb/IIIa, α-SMA and CD68 are shown. The primary antibody was substituted with the GP IIb/IIIa antigen-antibody complex as a preabsorption negative control. **(B)** The GP IIb/IIIa content in total plaque and coverage of the endothelium were quantified. Correlations between GP IIb/IIIa expression in plaques and **(C)** GP IIb/IIIa coverage of the endothelium, **(D)** plaque vulnerability index and **(E)** NC/FC ratio; and between GP IIb/IIIa coverage of the endothelium and **(F)** plaque vulnerability index and **(G)** NC/FC ratio were determined. *n* = 6 per group. Data represent the mean ± standard deviation. ^*^P <0.05 vs. C57BL/6 + HCD, ^#^*P* < 0.05 vs. ApoE ^−/−^ + NLD. α-SMA: α-smooth muscle actin; NC/FC: necrotic center/fiber cap.

To explore the relationship between GP IIb/IIIa expression and plaque vulnerability, GP IIb/IIIa expression in abdominal aortic plaques was detected in three experimental mouse groups (Figure 2A); H & E and Masson's trichrome staining, as well as CD68 and α-SMA immunolabeling were used to assess the plaque vulnerability index and the NC/FC ratio (Figure 2A); the quantitative results showed that both GP IIb/IIIa expression in plaques and coverage of the endothelium were highest in the ApoE^−/−^ + HCD group, followed by ApoE^−/−^ + NLD and C57BL/6 + HCD groups (*P* < 0.05; Figure 2B). Interestingly, GP IIb/IIIa expression in plaques were correlated with GP IIb/IIIa coverage of the endothelium (Figure 2C); GP IIb/IIIa expression in whole plaques and the coverage of the endothelium was also correlated with plaque vulnerability index and NC/FC ratio (Figures 2D–G).

**Figure 3:**
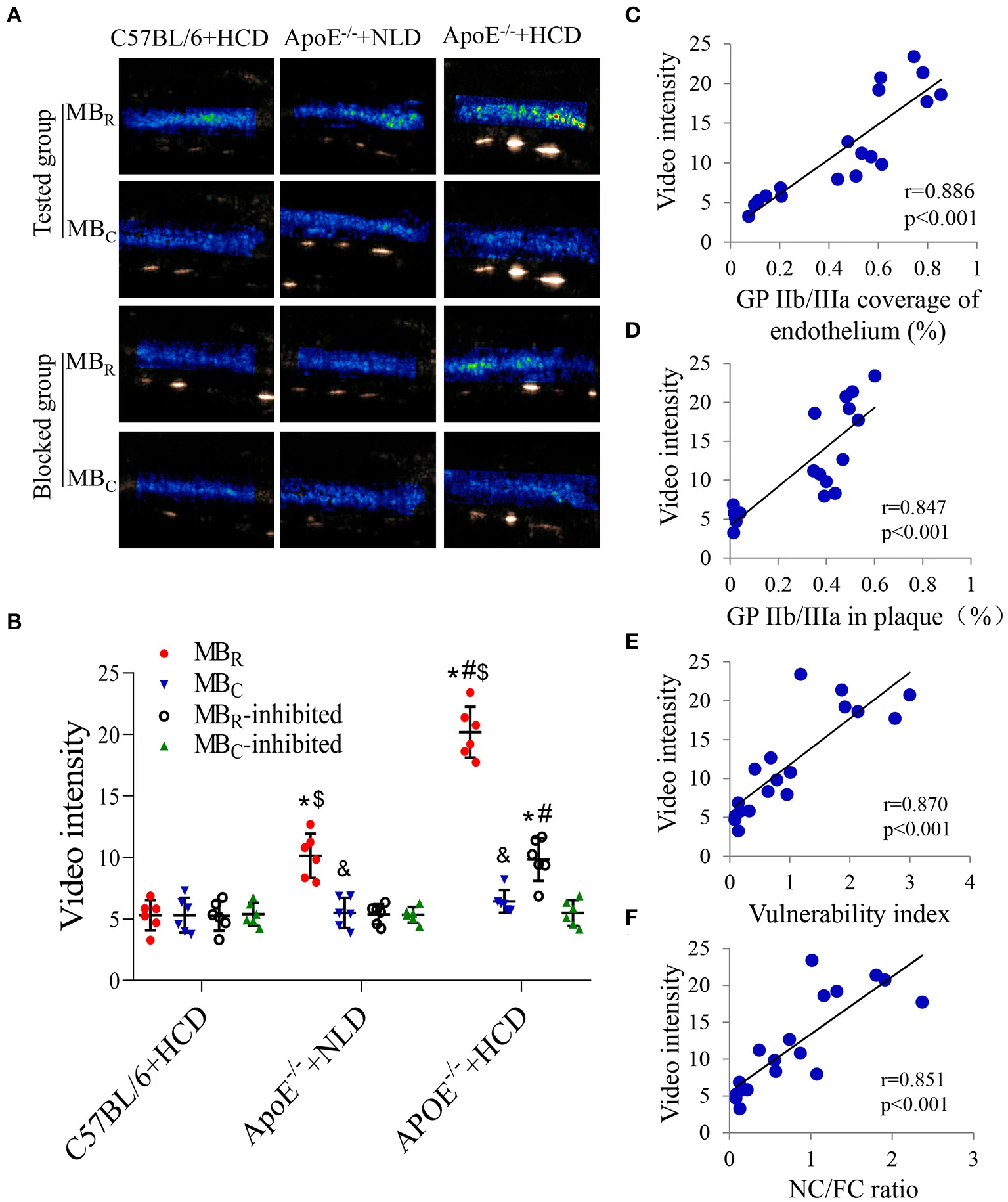
Correlations between video intensity of ultrasound molecular imaging and GP IIb/IIIa expression, and the plaque vulnerability indicators. **(A)** Background-subtracted, color-coded images were obtained after injection of MB_R_ or negative control (MB_C_). **(B)** Video intensity of MB_R_ and MB_C_ in the tested mice and their inhibited groups were quantified (*n* = 6 per group). Correlations between the video intensity of MB_R_ in tested group and **(C)** GP IIb/IIIa coverage of the endothelium, **(D)** GP IIb/IIIa expression in plaques, **(E)** the plaque vulnerability index, and **(F)** the NC/FC ratio are shown. *n* = 6 per group. Data represent the mean ± standard deviation. ^*^P < 0.05 vs. C57BL/6 + HCD, ^#^*P* < 0.05 vs. ApoE ^−/−^ + NLD, & *P* < 0.05, MB_C_ vs. MB_R_, ^$^*P* < 0.05, MB_R_ vs. MB_R_-inhibited. MB_R_: cyclic Arg-Gly-Asp (RGD)-modified microbubble; NC/FC: necrotic center/fiber cap.

In addition, the background-subtracted video intensity (Figure 3A) of MB_R_ and MB_C_ were comparable in both the tested and blocked groups of C57BL/6 + HCD mice, but the video intensity of MB_R_ were significantly higher than those of MB_C_ in the other two tested groups. The video intensity of MB_R_ in the tested group was highest in the ApoE^−/−^ + HCD group, followed by ApoE^−/−^ + NLD and C57BL/6 + HCD groups (*P* < 0.05; Figure 3B), with the similar trend as GP IIb/IIIa expression. The treatment with the GP IIb/IIIa antagonist decreased the video intensity to the level of MB_C_ in these groups (*P* < 0.05; Figure 3B), indicating that the binding between MB_R_ and GP IIb/IIIa receptor was mostly inhibited by Eptifibatide. Notably, the video intensity of MB_R_ in the tested group was correlated with GP IIb/IIIa coverage of the endothelium and expression in plaques (Figures 3C,D), and the plaque vulnerability index and NC/FC ratio (Figures 3E,F).

### The effects of DUMB_R_ treatment on plaques, skin and mesentery: Results of various ultrasonic MI values

**Figure 4:**
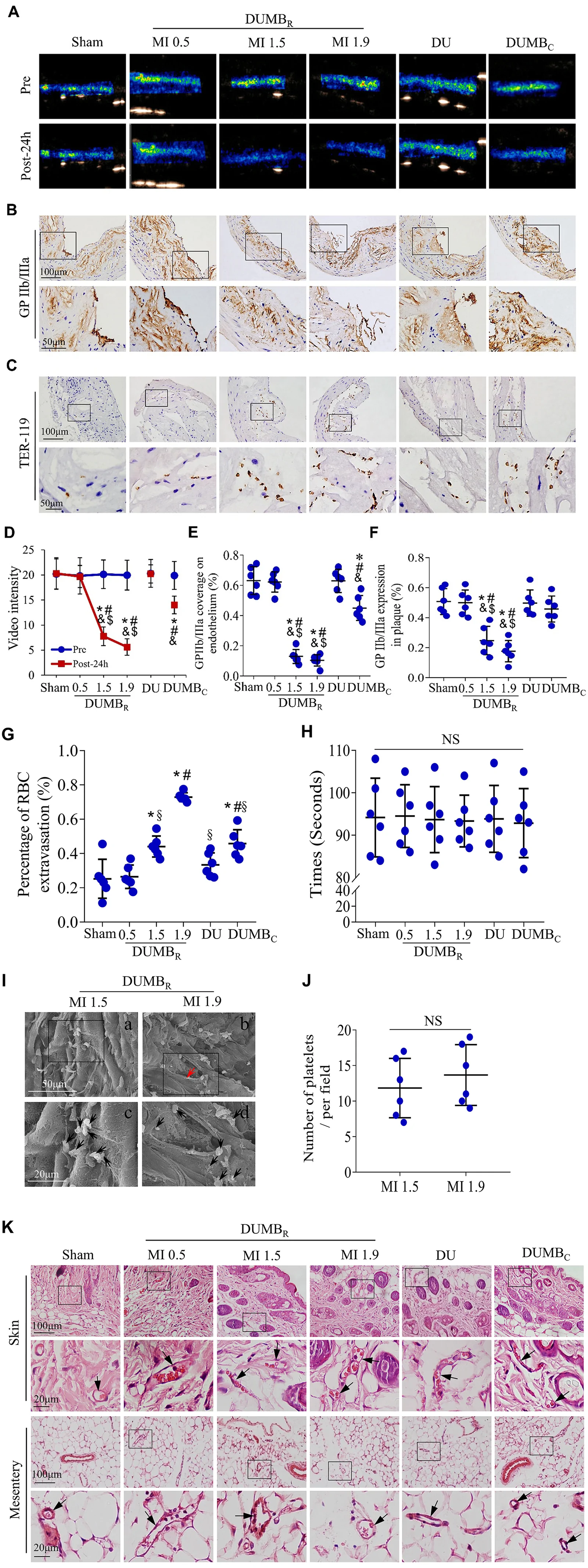
Effects of DUMB_R_ treatment on plaques: Results of various ultrasonic mechanical index (MI) values. **(A)** Background-subtracted, color-coded images of ultrasound molecular imaging for MB_R_ or MB_C_ before and 24 h after treatment. Representative images of immunohistochemical staining for **(B)** GP IIb/IIIa and **(C)** TER-119 in plaques are shown. **(D)** The video intensity of MB_R_ or MB_C_ in mice was quantified. Quantitative analysis of the **(E)** GP IIb/IIIa coverage on the endothelium, **(F)** GP IIb/IIIa expression in plaques, and **(G)** the average number of red blood cells at 24 h after treatment. **(H)** Bleeding times were assessed by surgical tail transection in the six treated mouse groups (*P* > 0.05). **(I)** Platelets (black arrowheads) adhering to the site of atherosclerotic lesions were observed by scanning electron microscopy after the DUMB_R_ treatment at an MI of 1.5 or 1.9 groups. Panel b and its magnified images in panel d show that the endothelium was incomplete (red arrowheads). **(J)** Average number of activated platelets was quantified. **(K)** Representative images of abdominal skin and mesentery stained with H & E. Red blood cells were observed in complete vessels (black arrowheads). *n* = 6 per group. ^*^P < 0.05 vs. sham, ^#^*P* < 0.05 vs. MI 0.9, ^&^*P* < 0.05 vs. DU only, ^$^*P* < 0.05 vs. DUMB_C_, ^§^*P* < 0.05 vs. MI 1.9 DUMB_R_; DUMB_R_: diagnostic ultrasound combined with MB_R_. DUMB_C_, diagnostic ultrasound combined with MB_C_.

Ultrasound molecular imaging was used to reveal the video intensity of MB_R_ or MB_C_ in the abdominal aorta in different treatment mouse groups before and 24 h after DUMB_R_ treatment (Figure 4A). Before treatment, the video intensity were comparable among the six different treatment mouse groups, indicating equivalent plaque vulnerability in these mice (*P* > 0.05; Figure 4D). 24 h after treatment, the video intensity were comparable in the sham group, MI 0.5 DUMB_R_-treated group and DU-only group and were slightly higher than those in the DUMB_C_-treated group, while the video intensity in the MI 1.5 and MI 1.9 DUMB_R_ treatment groups were significantly decreased compared with those in the other four groups (*P* < 0.05; Figure 4D).

GP IIb/IIIa immunostaining was used to assess the activated and aggregated platelets in abdominal aortic plaques in different treatment groups 24h after treatment (Figure 4B), the ratio of GP IIb/IIIa coverage of the endothelium did not significantly differ among the sham, MI 0.5 DUMB_R_-treated and DU-only groups, but was slightly decreased in DUMB_C_-treated group, and further decreases were observed in the MI 1.5 and MI 1.9 DUMB_R_-treated groups compared with the sham, MI 0.5-treated and DU-only groups (*P* < 0.05; Figure 4E). A similar expression pattern was observed in plaques in these groups (*P* < 0.05; Figure 4F). Notably, the plaque endothelium was destroyed in the MI 1.9 DUMB_R_-treated group compared with that in the other groups. in addition, we further used scanning electron microscopy to observe the activated platelets adhered to the surface of the vessel lumen in the MI 1.5 and MI 1.9 DUMB_R_ treatment groups. there was no significant difference in the number of activated platelets between the two groups (*P* > 0.05; Figures 4I,J), but the structures of plaque endothelium were indeed incomplete in the MI 1.9 DUMB_R_ Group compared with those in the MI 1.5 DUMB_R_ group (Figure 4I). In addition, TER-119 immunostaining was used to reveal the extravasation of erythrocytes within plaques in different treatment groups 24 h after treatment (Figure 4C). The number of exosmotic erythrocytes was most significantly increased in the MI 1.9 DUMB_R_ Group, followed by the DUMB_C_ Group, MI 1.5 and 0.5 DUMB_R_ Groups, the DU only group and sham group (*P* < 0.05; Figure 4G). Moreover, Supplementary Figure 1 demonstrated that expression of TER-119 was slightly increased at 24 h after treatment, but significantly decreased at 8 Weeks and 12 Weeks after DUMB_R_ treatment at an MI of 1.5 compared with pretreatment (*P* < 0.05). These indicate that DUMB_R_ treatment did not further increase erythrocyte extravasation within plaques and MI 1.5 may be a safer ultrasonic condition for DUMB_R_ treatment of atherosclerotic plaque.

H & E staining of abdominal skin and the mesentery was used to evaluate the influence of DUMB_R_ treatment on surrounding normal tissue. No significant extravasated erythrocytes or, degeneration or necrosis of cells was found in the skin and mesentery after DUMB_R_ treatment at different MI values or after DU only treatment or DUMB_C_ treatment groups (Figure 4K). Furthermore, the bleeding time were assessed by surgical tail transection, there were no differences in bleeding times among the six treatment mouse groups (*P* > 0.05, Figure 4H). Thus, DUMB_R_ treatment at different MI values does not compromise normal tissue surrounding the abdominal aorta and prolonging the bleeding time.

### Targeted dissolution of activated platelets aggregated on plaque

**Figure 5:**
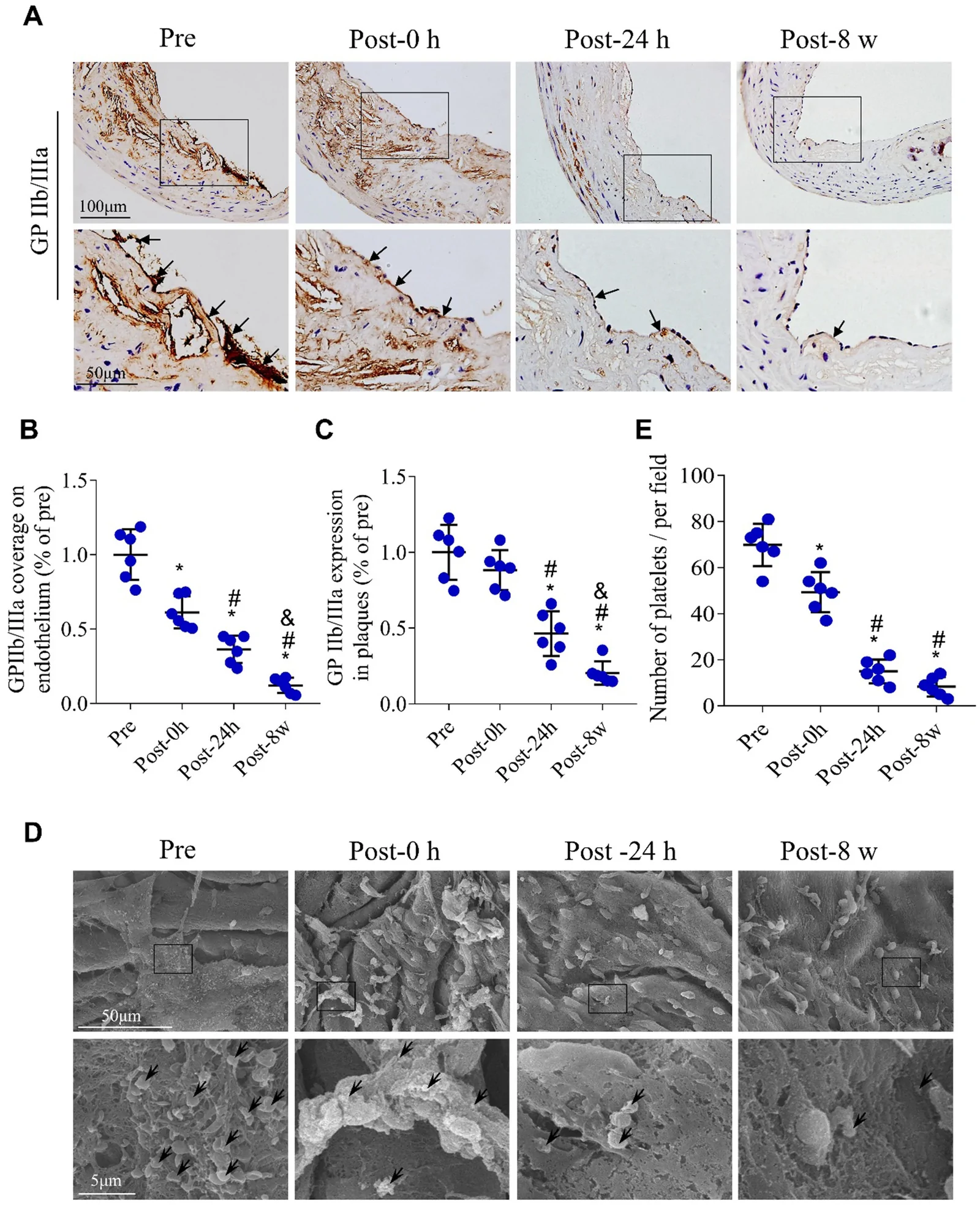
Effects of DUMB_R_ treatment at an MI of 1.5 on activated platelets in plaques. **(A)** Representative images of GP IIb/IIIa labeling in plaques and the endothelium (arrows) of the aorta are shown. Quantifications of **(B)** GP IIb/IIIa coverage of the endothelium and **(C)** total plaque GP IIb/IIIa content are shown. **(D)** Platelets (black arrowheads) adhering to the endothelium of atherosclerotic lesions were observed by scanning electron microscopy. **(E)** Quantitative analysis of the average number of activated platelets. *n* = 6 per group. ^*^P < 0.05 vs. pretreatment, ^#^*P* < 0.05 vs. Post-0 h, ^&^*P* < 0.05 vs. Post-24 h.

Immunohistochemistry of GP IIb/IIIa was performed at the four time points to assess the activated platelets aggregated on plaque (Figure 5A). Compared with pretreatment, GP IIb/IIIa coverage of the endothelium was significantly decreased at 0 h, 24 h, and 8 weeks after DUMB_R_ treatment (*P* < 0.05; Figure 5B), and a similar expression trend was observed in plaques (*P* < 0.05; Figure 5C). In addition, we further used scanning electron microscopy to detect the activated platelets adhered on the surface of plaques in the groups at four time points (Figure 5D). The number of activated platelets aggregated on the endothelium showed a trend similar to that of GP IIb/IIIa expression (Figure 5E).

### DUMB_R_ treatment reduces the expression of platelet adhesion promoters, thrombogenic factors, adhesion molecules, chemokines and microvasculature in plaques

**Figure 6:**
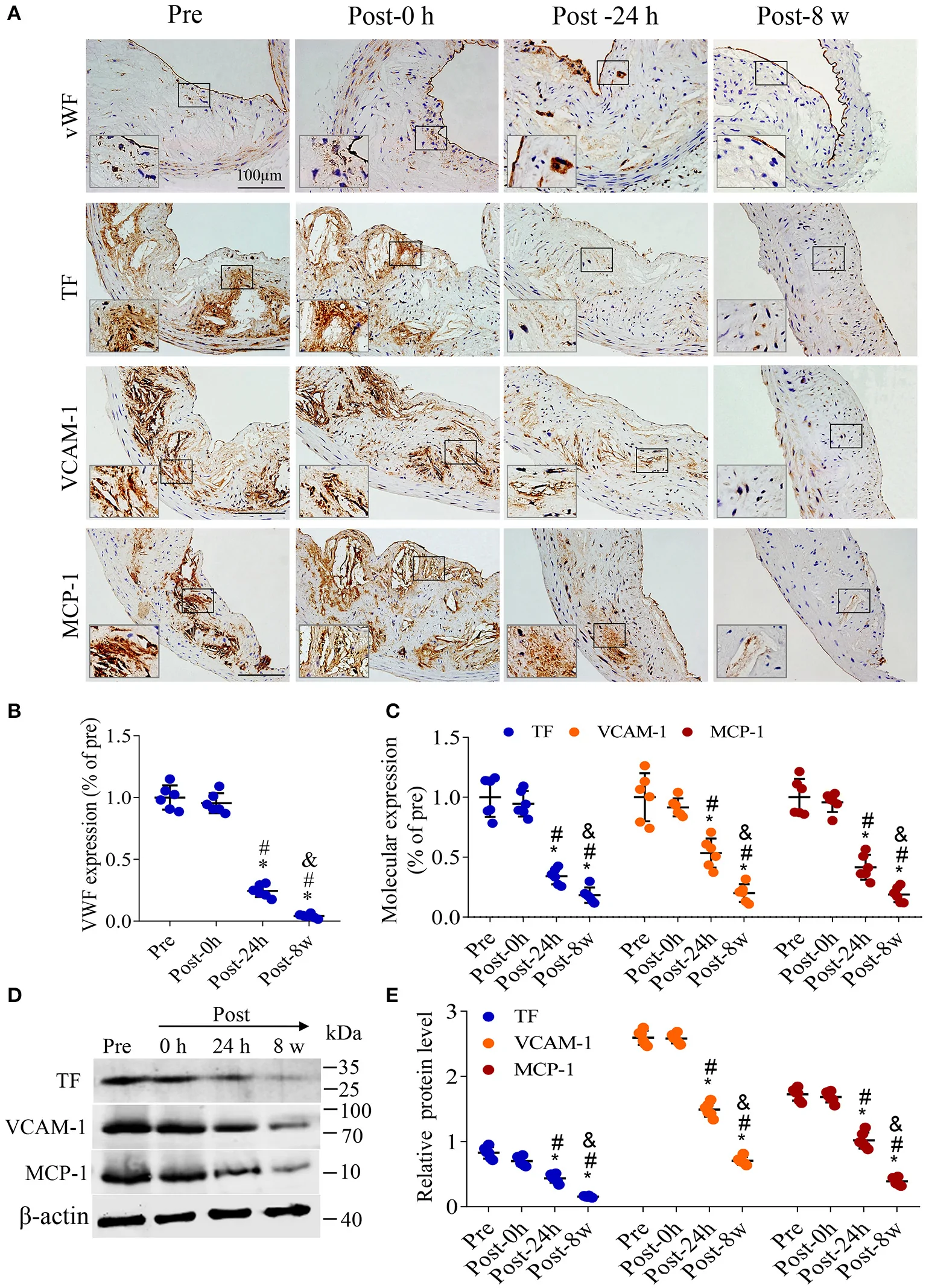
DUMB_R_ treatment reduces the expression of platelet adhesion promoters, thrombogenic factors, adhesion molecules and chemokines. **(A)** Representative images of Immunohistochemistry for von Willebrand factor (vWF), tissue factor (TF), vascular cell adhesion molecule-1 (VCAM-1) and monocyte chemotactic protein (MCP-1) before treatment and at 0 h, 24 h and 8 weeks after treatment with DUMB_R_ at an MI of 1.5. Quantification of **(B)** vWF, **(C)** TF, VCAM-1 and MCP-1 at different time points is shown. **(D)** Representative immunoprecipitation images of TF, VCAM-1 and MCP-1 in plaques before treatment and at 0 h, 24 h, and 8 w after treatment with DUMB_R_ at an MI of 1.5. **(E)** Quantification of the immunoblot strip. *n* = 6 per group. ^*^P < 0.05 vs. pretreatment. ^#^*P* < 0.05 vs. Post-0 h, & *P* < 0.05 vs. Post-24 h, *n* = 6 per group.

To explore the possible mechanism of plaque instability improvement, the expression of vWF, TF, VCAM-1 and MCP-1 in the plaques is shown by immunohistochemistry in Figure 6A. No significant difference in the expression of the platelet adhesion promoter (represented by vWF) was observed immediately after DUMB_R_ treatment compared with pretreatment; however, at 24 h and 8 weeks after treatment, the expression of vWF was significantly decreased (*P* < 0.05; Figures 6A,B). Similar patterns were observed in the expression of thrombogenic factors, endothelial adhesion molecules and macrophage chemokines (*P* < 0.05; Figures 6A,C); consistently, the expression of TF, VCAM-1 and MCP-1 by western-blotting also presented similar expression trends (*P* < 0.05; Figures 6D,E). In addition, plaque microvasculature (marked by CD31) were dramatically decreased at 24 h, and 8 weeks after treatment (*P* < 0.05; Supplementary Figures 2A,B).

### DUMB_R_ treatment improves plaque instability

**Figure 7:**
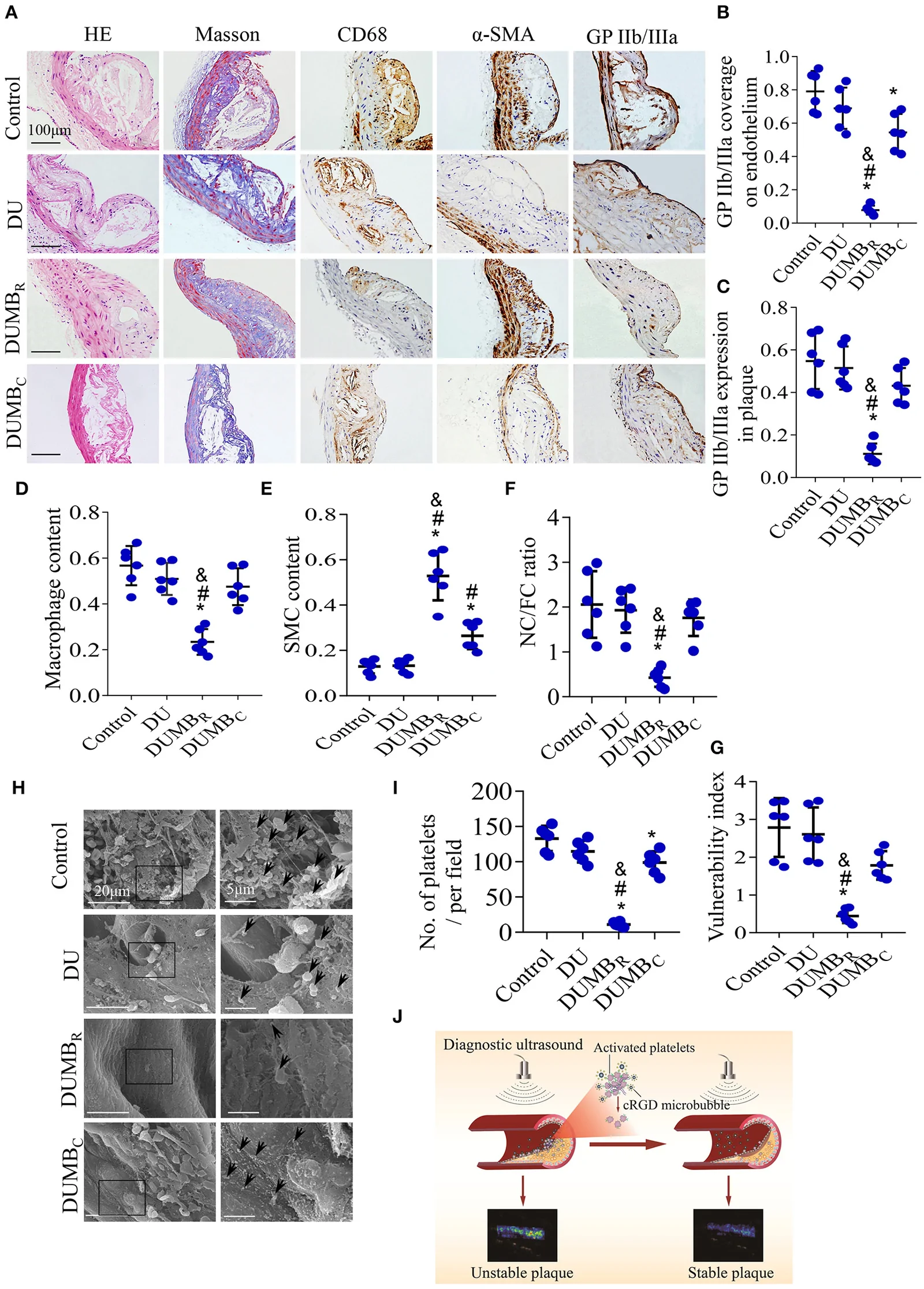
Effect of DUMB_R_ treatment on plaque instability. **(A)** Representative images of H & E and Masson's trichrome staining and immunolabeling with antibodies against α-SMA, CD68 and GP IIb/IIIa. Quantitative analyses of **(B)** GP IIb/IIIa coverage of the endothelium, **(C)** total plaque GP IIb/IIIa content, **(D)** plaque macrophage and **(E)** SMC content were shown. **(F)** The plaque NC/FC ratio and **(G)** vulnerability index were calculated. **(H)** Representative scanning electron microscopy images of activated platelets (black arrowheads). **(I)** Average number of activated platelets was quantified. **(J)** Illustration of DUMB_R_ identifying unstable plaques and simultaneously improving their stability by specifically dissolving activated platelets. n = 6 per group. *P < 0.05 vs. control, #P < 0.05 vs. DU, &P < 0.05 vs. DUMB_C_. DUMB_R_, diagnostic ultrasound combined with MB_R_; DUMB_C_, diagnostic ultrasound combined with MB_C_.

Twelve weeks after DUMB_R_ treatment, H & E and Masson's trichrome staining, as well as CD68, α-SMA and GP IIb/IIIa immunolabeling of abdominal aortic plaques (Figure 7A), revealed that both GP IIb/IIIa expression in plaques and coverage of the endothelium were markedly reduced in the DUMB_R_ treatment group, followed by DUMB_C_ treatment, DU only and control groups. Similar trends were observed in the abundance of macrophages, the vulnerability index and NC/FC ratio (*P* < 0.05; Figures 7B–D,F,G). In contrast, the expression of SMCs showed the opposite trend (Figure 7E). Additionally, the results of scanning electron microscopy also indicated that at 12 weeks after DUMB_R_ treatment, compared to the DUMB_C_ treatment and other two groups, fewer activated platelets adhered and aggregated on the endothelium (Figures 7H,I), which is consistent with the expression of GP IIb/IIIa described above. In summary, DUMBR could identify unstable plaques and simultaneously improve their stability by specifically dissolving activated platelets as illustrated in Figure 7J.

## Discussion

The present study demonstrated that DUMB_R_ targeting GP IIb/IIIa receptors could simultaneously and specifically diagnose and treat atherosclerotic plaques and evaluate the treatment effect. The underlying mechanism of stabilizing atherosclerotic plaques may be due to the targeted dissolution of activated and aggregated platelets, with a reduction in vWF expression, which further reduces the thrombogenic risk, vascular inflammation, and macrophage recruitment, and ultimately enhances plaque stability. This theranostic approach combining diagnostic and therapeutic capabilities in a single type of targeted MB with high specificity and affinity may represent a novel application for the detection and stabilization of atherosclerotic plaques while avoiding systemic adverse effects.

A key contributor to thrombosis, activated platelets could be considered a critical factor leading to plaque instability and rupture, and the GP IIb/IIIa receptor of activated platelets has been regarded as a target for the diagnosis of high-risk plaques (3, 4). This was supported by our finding that GP IIb/IIIa coverage of the endothelium or expression in plaques was correlated with both plaque vulnerability index, the ratio of NC/FC and video intensity of ultrasound molecular imaging for MB_R_ on plaques. Moreover, our results also revealed that the video intensity of MB_R_ in plaques was comparable at baseline, while a significant reduction was observed in the MI 1.5 and 1.9 DUMB_R_-treated groups, which was mainly due to the platelet aggregated clot disruption caused by ultrasound-MB_R_ cavitation effect. This indicates that DUMB_R_ could detect the consistency of the plaque features before treatment and reliably evaluating the therapeutic efficacy after treatment, which is consistent with previous reports (4, 11).

As a critical biomarker of plaque instability, GP IIb/IIIa receptor on activated platelets in plaques has also been proposed as a key target for the treatment of advanced atherosclerotic plaques (14). Accumulating evidence suggests that effective antiplatelet drug therapy, affecting the process of platelet activation, adhesion or aggregation can stabilize atherosclerotic plaques but yields a limited reduction in fatal acute events and an increased risk of bleeding complications after systemic administration (6, 17, 23). Additionally, pioneering groups have demonstrated that DU+MB can dissolve acute intravascular thrombi in large vessels and platelet-rich microthrombi in small vessels or microvessels both *in vitro* and *in vivo* (9, 10). However, the application of such modalities to atherosclerotic plaques is still limited, and to date, the effect of DUMB_R_ treatment on plaque instability and the underlying mechanism have not been explored. In the present study, the particle size distribution of MBs results show that the MBs contain not only micron-sized MBs but also a large number of nano-sized MBs, which could penetrate into the neovascular wall through the microvascular space (24), allowing more MBs to enter the atherosclerotic plaque and reach the activated platelets in the plaque. Hence, DUMB_R_ can effectively dissolve activated and aggregated platelets in atherosclerotic plaques. The results of immunohistochemistry and scanning electron microscopy illustrate that DUMB_R_ at an ultrasound MI of 1.5 significantly reduced GP IIb/IIIa expression coverage on endothelia and within plaques and the number of activated platelets on endothelia. This phenomenon could be explained by the dissolution of aggregated platelets by ultrasound-medicated destruction of MB_R_. More importantly, our results also indicate that dissolving aggregated platelets by DUMB_R_ significantly improved plaque instability. After treatment with DUMB_R_ at an MI of 1.5, the SMC content in the plaques was obviously increased, while the macrophage content, the corresponding vulnerability index and the NC/FC ratio were significantly decreased, thereby improving plaque instability. Therefore, DUMB_R_ targeting GP IIb/IIIa receptors can effectively dissolve activated and aggregated platelets in plaques and improve the instability of atherosclerotic plaques.

It is widely believed that DUMB_R_ at an appropriate MI can confer excellent antiplatelet efficacy and has minor side effects. Ultrasonic MI is one of the most important parameters that determine the conditions of ultrasound molecular imaging or targeted therapy (25). To determine the most suitable MI parameters, a series of factors, such as the video intensity of ultrasound molecular imaging of MB_R_, activated platelets and erythrocyte extravasation in plaque, were observed at four different levels of ultrasonic MI with or without MB_R_. Our results indicate that the video intensity of the abdominal aorta gradually decreased as the ultrasonic MI increased, reaching a plateau at 1.5 and did not further decrease even when the MI was increased to 1.9 with MB_R_ or MB_C_. This finding was further supported by the GP IIb/IIIa immunostaining and scanning electron microscopy results. Nonetheless, the endothelium of the plaque was destroyed in the group treated with DUMB_R_ at an MI of 1.9 compared with that in the other treatment groups as observed by GP IIb/IIIa immunostaining. Additionally, the scanning electron microscopy results also showed that the structure of the plaque endothelium was incomplete in the MI 1.9 DUMB_R_ group compared with that in the MI 1.5 DUMB_R_ group. This finding indicates that the DUMB_R_ treatment at an MI of 1.5 did not increase the risk of endothelial damage but had a therapeutic effect similar to that of the MI 1.9 group, which is consistent with the observation of a positive relationship between MB-mediated ultrasound cavitation intensity and vascular endothelial damage (26). In addition, our TER-119 immunostaining results revealed that the extravasation of erythrocytes within plaques was more significantly increased in the MI 1.9 DUMB_R_ group compared to that in the DUMB_C_ and MI 1.5 DUMB_R_ groups and the other groups. This finding indirectly indicated that DU at higher MI values with MB_R_ may induce the fibrous cap rupture or intraplaque hemorrhage resulting from capillary damage in high-risk atherosclerotic plaques (20), which requires careful attention. Previous studies demonstrated that both MB destruction and the number of microvascular ruptures were proportional to the applied MI, which just confirmed our findings (20, 26, 27). Notably, our supplementary results show that immunostaining for TER-119 did not further increase after DUMB_R_ treatment at an MI of 1.5 and continued to decrease over time. In this study, we also found that the DUMB_R_ at an MI of 1.5 did not prolong the bleeding time. Therefore, these above indicate that DUMB_R_ treatment at an MI of 1.5 is more appropriate and safer for targeted dissolution of activated and aggregated platelets and has fewer adverse effects on normal tissues.

Once an atherosclerotic vulnerable plaque suddenly disrupts or erodes, the subendothelial matter is exposed to circulating blood components, leading to platelet activation and subsequent thrombus formation; endotheliogenic vWF first initiates platelet adhesion onto the endothelium *via* an interaction its A1 domain and the GP Ib-V-IX receptor in the initial phase; then induces the activation of platelet GP IIb/IIIa receptor through an “inside-out” conformational change that switches to a high-affinity state and finally permits the activation of TF in the subsequent enlarged stage (14, 28, 29). Thereafter, TF binds activated coagulation factor VII and recruits the circulating platelets and inflammatory cells, resulting in a vicious cycle of “platelet activation-thrombogenesis” (29, 30). Hence, dissolution of aggregated platelets by DUMB_R_, which reduces the number of activated platelets and the levels of related mediators, may interrupt this cycle and reduce platelet activation and thrombus formation. Our current immunohistochemical results show that the expression of vWF at 24 h and 8 weeks after DUMB_R_ treatment was significantly reduced compared with that in the pretreatment group. Moreover, the levels of TF were markedly decreased, indicating that DUMB_R_ could decrease the risk of thrombus formation. In addition, the content of adhesion molecules and macrophage infiltration, which could aggravate the inflammatory response, causing plaque instability or rupture, was also significantly decreased. The same phenomenon was reflected in the western blotting data. In addition, in our study, both plaque vulnerability and atherosclerotic lesion areas were markedly decreased after the treatment with DUMB_R_.These finding indicate that DUMB_R_ treatment could reduce vWF expression, which further reduces the thrombogenic risk, vascular inflammation, and macrophage recruitment, and ultimately improve plaque instability. Furthermore, our supplementary results show that plaque microvasculature declined over time after the DUMB_R_ treatment which may further enhances plaque stability. In summary, the DUMB_R_ strategy in current study could specifically identify, target, and dissolve aggregated platelets and then evaluate the therapeutic efficacy in real time after treatment. It is exciting that a strategy combining DU with MB has been applied to the dissolution of acute platelet-rich thrombi in mouse, rat and canine models (10, 11, 31), highlighting the potential for future application in atherosclerotic plaques in humans but needed to be investigated by further clinical trials. Therefore, the DUMB_R_ approaches used in the current study, which could provide targeted identification and dissolution of aggregated platelets in plaques to avoid systemic side effects, may provide a novel strategy for detecting and stabilizing atherosclerotic plaques.

There are several limitations of our study. First, the amount of GP IIb/IIIa on activated platelets is much greater than that of other integrins, such as αvβ3, αvβ1, and αvβ6. However, these integrins can also bind MB_R_ similar to GP IIb/IIIa, contributing to an enhanced ultrasound signal for MB_R_, which is weaker in the presence of a GP IIb/IIIa inhibitor; thus, we can assume that the signal of MB_R_ was mainly derived from GP IIb/IIIa of activated platelets on plaques. An alternative approach could make apply MB with a GP IIb/IIIa-specific antibody, but immunogenicity may limit its clinical applications (4, 15, 32). Second, we did not observe in real-time whether the platelet aggregates returned at other time points within 12 weeks or longer after a single DUMB_R_ treatment. However, GP IIb/IIIa expression and the number of platelets aggregates were indeed significantly decreased at 24 h, 8 weeks and 12 weeks after treatment, and the DUMB_R_ treatment group was more stable than the other groups at 12 weeks, which was consistent with previous studies (20). Third, similar to previous studies (33), only a small fraction of the atherosclerotic plaques that were generated could be defined as vulnerable plaques, which could cause severe cardiovascular events; however, plaque vulnerability was observed in many mice, especially those fed an HCD, as evidenced by the atherosclerotic vulnerable indicators. Moreover, the technology has never been applied for the intracranial plaques, but multiple studies have proved that certain intensity of ultrasound could achieve the penetration of the skull and application on intracranial microthrombi (10, 34), which would provide a potential adjunct to therapy of intracranial plaques. Furthermore, our study was performed in a mouse model, which may not completely reflect all aspects of atherosclerotic plaques in humans, although the ultrasonic parameters used in the present study have potential value for clinical applications, further studies verifying the results in larger animals or humans could rapidly promote the translation of this novel theranostic method to clinical application, which is the goal of our future research.

## Conclusion

In conclusion, the data in this study indicate that DU combined with MB_R_ targeting GP IIb/IIIa receptors could simultaneously and selectively identify and stabilize atherosclerotic plaques by imaging and dissolving aggregated platelets. Therefore, DUMB_R_ technology combining diagnostic and therapeutic capabilities using a single type of targeted MB may represent a novel application for the simultaneous detection and therapy of atherosclerotic plaque.

## Data availability statement

The raw data supporting the conclusions of this article will be made available by the authors, without undue reservation.

## Ethics statement

The animal study was reviewed and approved by Institutional Animal Care and Use Committee of Zhengzhou University.

## Author contributions

FH and SG conceived and designed the experiments. SG, SZ, and XC performed the experiments. SG, KC, and FH analyzed and interpreted the data. FH, SG, SZ, and KC drafted the manuscript and critically assessed its contents. All authors have read and approved the final version of the manuscript, have made important contributions to the study, and are familiar with the data.

## Funding

This study was supported by the National Natural Science Foundation of China (No. 81701695) and Youth Foundation of the First Affiliated Hospital of Zhengzhou University (No. YNQN2017118). This study was also supported by the Medical Science and Technology Research Project of Henan Province (Grant No. SBGJ202102153) and Henan Provincial Science and Technology Research Projects (Grant Nos. 212102310799 and 222102310577).

## Conflict of interest

The authors declare that the research was conducted in the absence of any commercial or financial relationships that could be construed as a potential conflict of interest.

## Correction note

A correction has been made to this article. Details can be found at: 10.3389/fcvm.2026.1943413.

## Publisher's note

All claims expressed in this article are solely those of the authors and do not necessarily represent those of their affiliated organizations, or those of the publisher, the editors and the reviewers. Any product that may be evaluated in this article, or claim that may be made by its manufacturer, is not guaranteed or endorsed by the publisher.

## Abbreviations

MB_R_: cyclic Arg-Gly-Asp (RGD)-modified microbubble
ApoE^−/−^: apolipoprotein E-deficient
HCD: hypercholesterolemic diet
DUMB_R_: diagnostic ultrasound combined with MB_R_
MI: mechanical index
MB_C_: negative control MB
α-SMA: α-smooth muscle actin
SMCs: smooth muscle cells
vWF: von Willebrand factor
TF: tissue factor
MCP-1: monocyte chemotactic protein
VCAM-1: vascular cell adhesion molecule-1
NC/FC: necrotic center/fiber cap.
