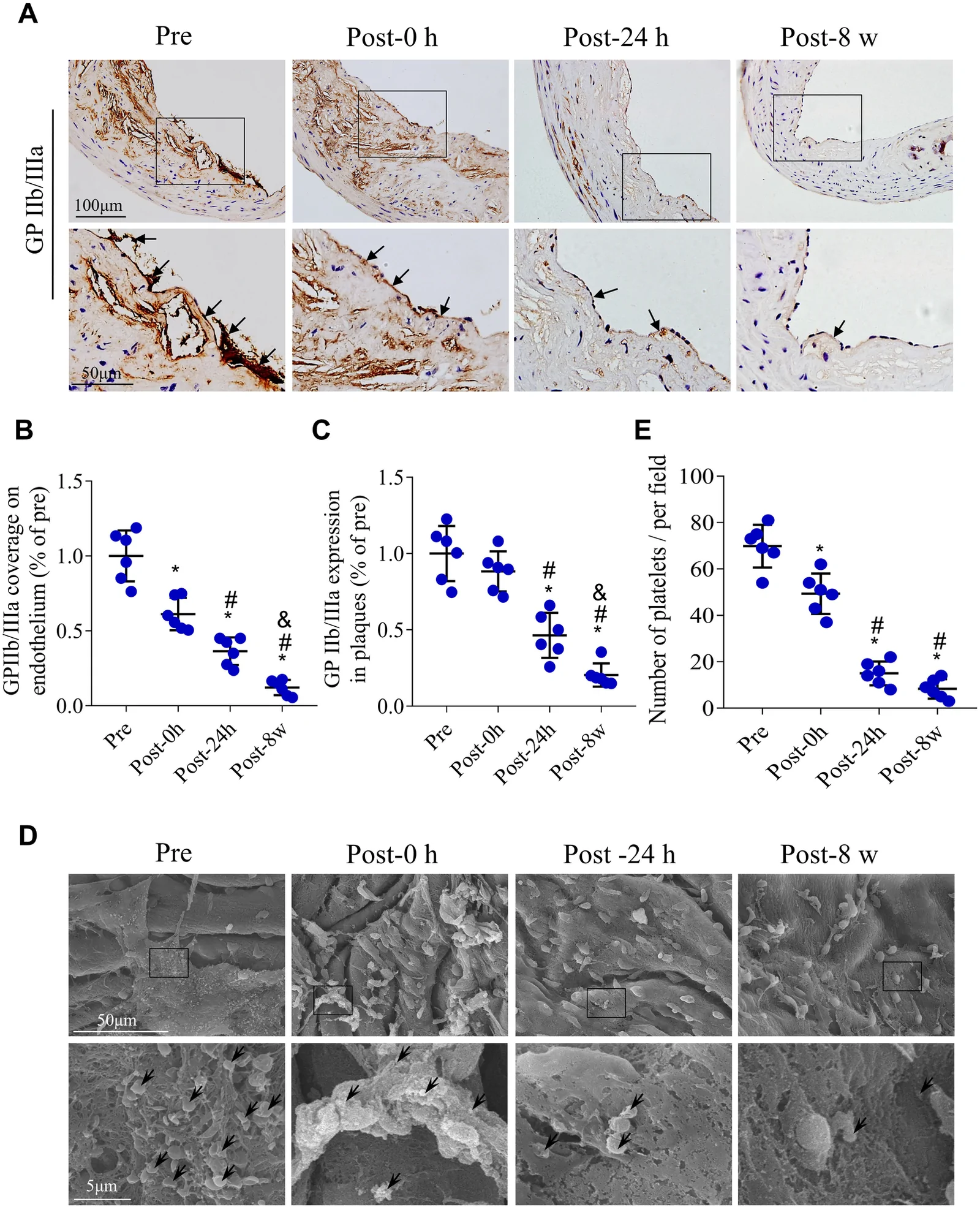# Correction: Effects of diagnostic ultrasound with cRGD-microbubbles on simultaneous detection and treatment of atherosclerotic plaque in ApoE^−/−^ mice

**DOI:** 10.3389/fcvm.2026.1943413

**Published:** 2026-10-09

**Authors:** Shengcun Guo, Shengye Zhang, Kui Chen, Xi Chen, Fudong Hu

**Affiliations:** Department of Cardiology, The First Affiliated Hospital of Zhengzhou University, Zhengzhou, China

**Keywords:** atherosclerotic plaque, diagnostic ultrasound, microbubbles, activated platelets, GP IIb/IIIa receptor

The published versions of Figures 4K and 5A contained unintentional formatting errors introduced during figure compilation. Calibrated scale bars were also absent from the original Figure 4K panel. The corrected Figure 4K now includes clearly marked, calibrated scale bars to support accurate morphological quantification. These corrections are limited to figure layout and presentation; the original raw data, statistical analyses, quantitative results, and study conclusions remain unchanged. The corrected Figures 4K and 5A are shown below.

The original version of this article has been updated.

**Figure 4:**
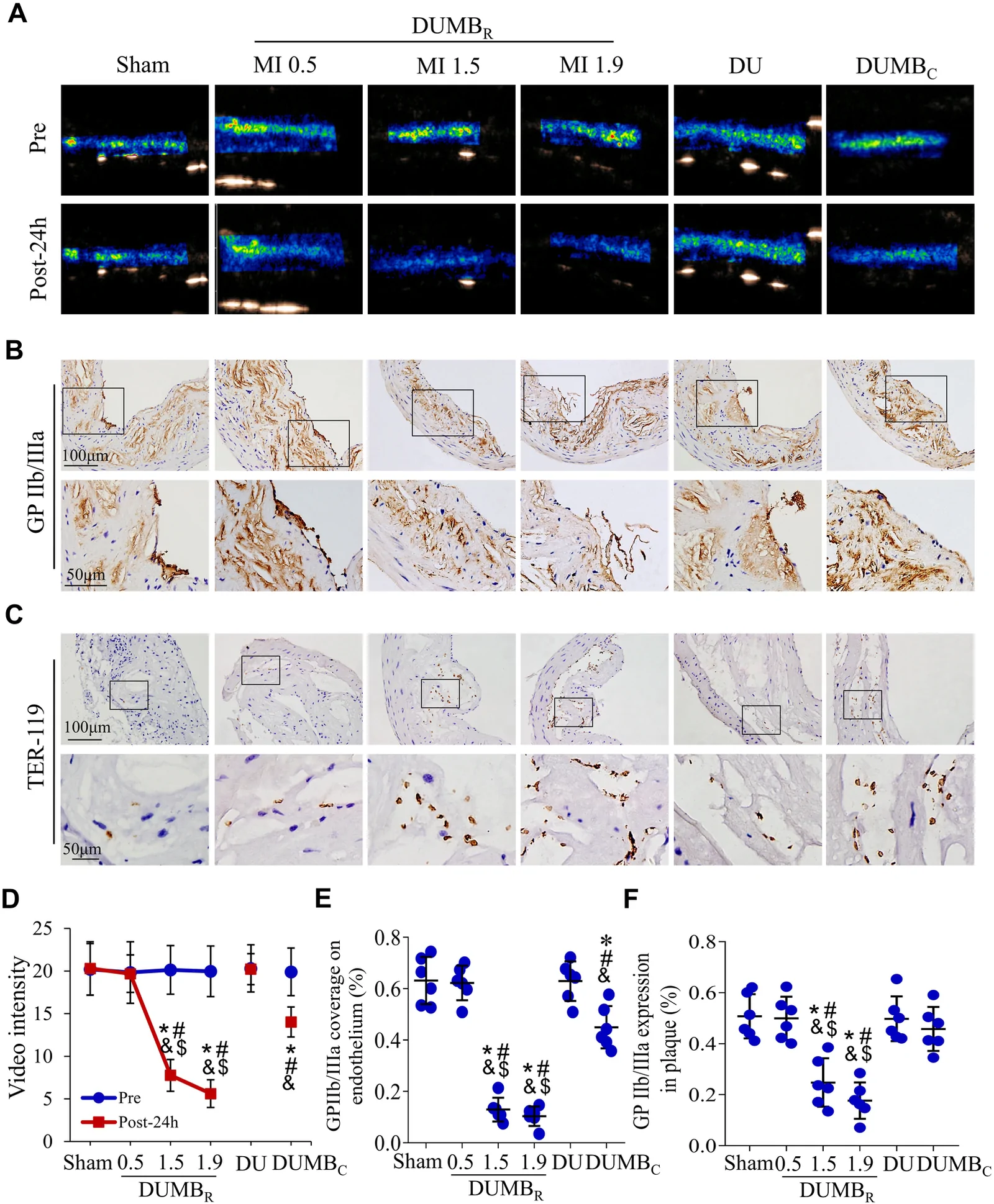

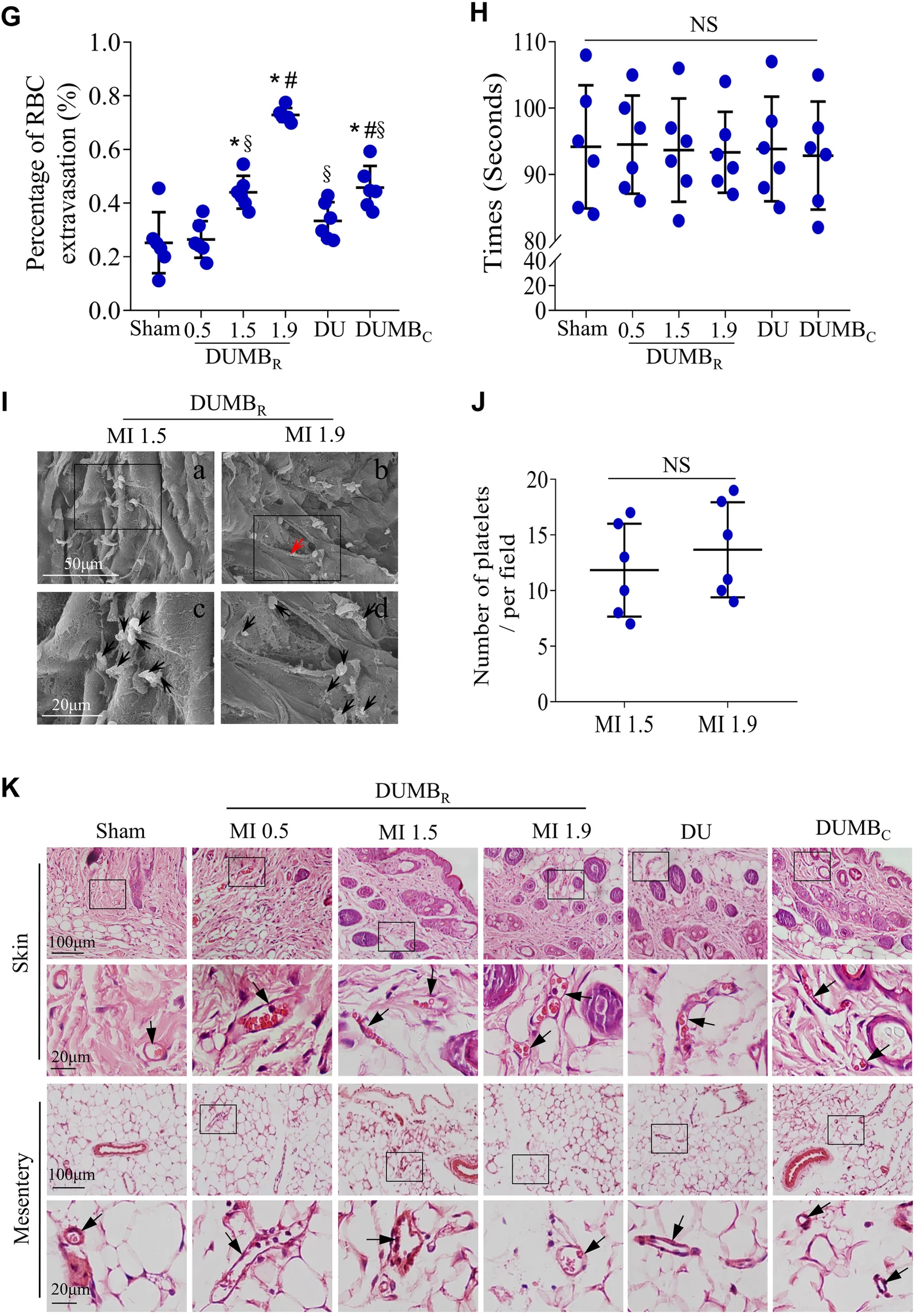


**Figure 5:**