# Supplementary figures and images for: Effects of diagnostic ultrasound with cRGD-microbubbles on simultaneous detection and treatment of atherosclerotic plaque in ApoE−/− mice
Source: Front Cardiovasc Med. 2022 Jul 22;9:946557. doi: 10.3389/fcvm.2022.946557 (PMC9354833; doi:10.3389/fcvm.2022.946557)

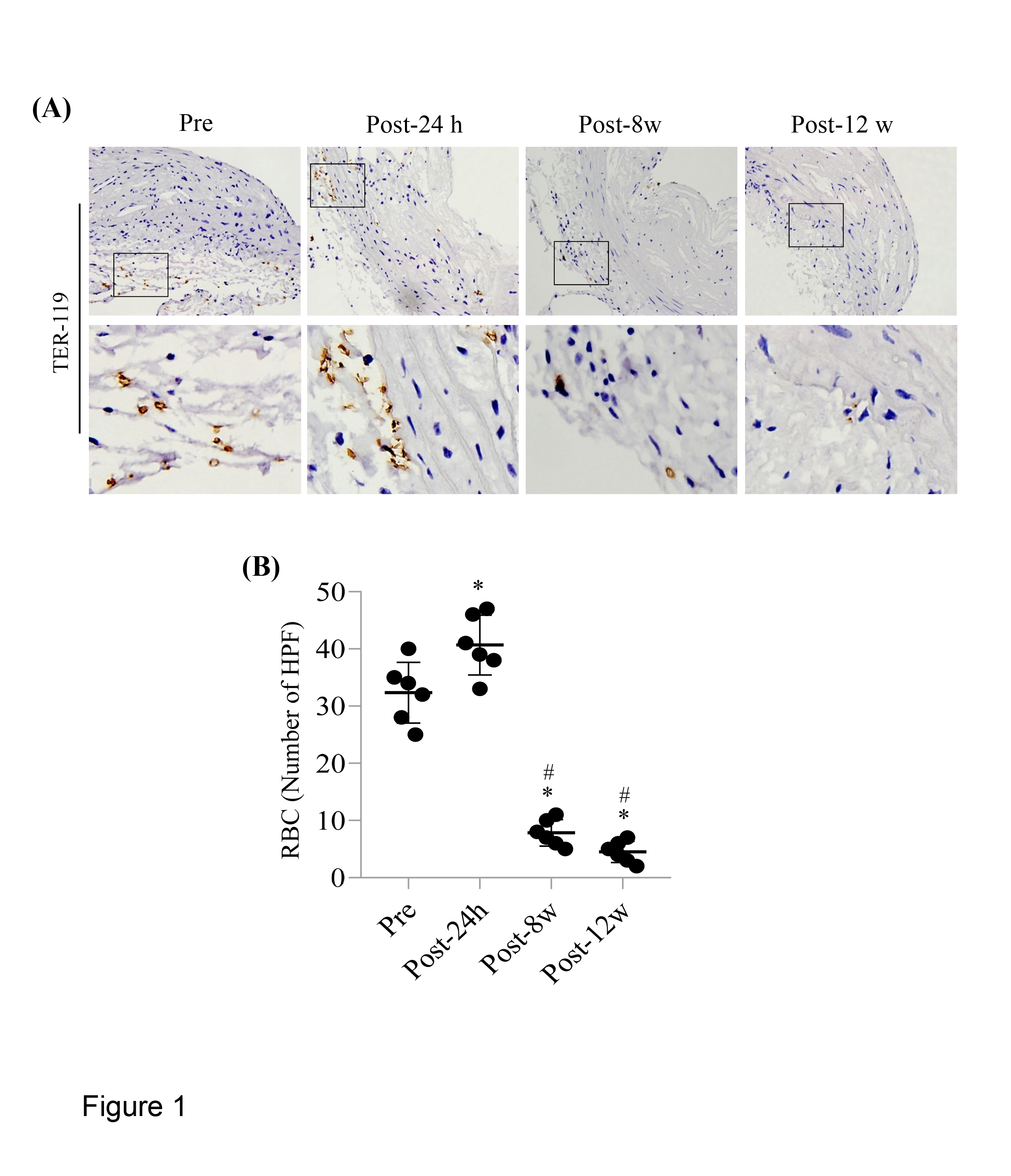

Supplement: Supplementary file 1 [file Image_1.JPEG]

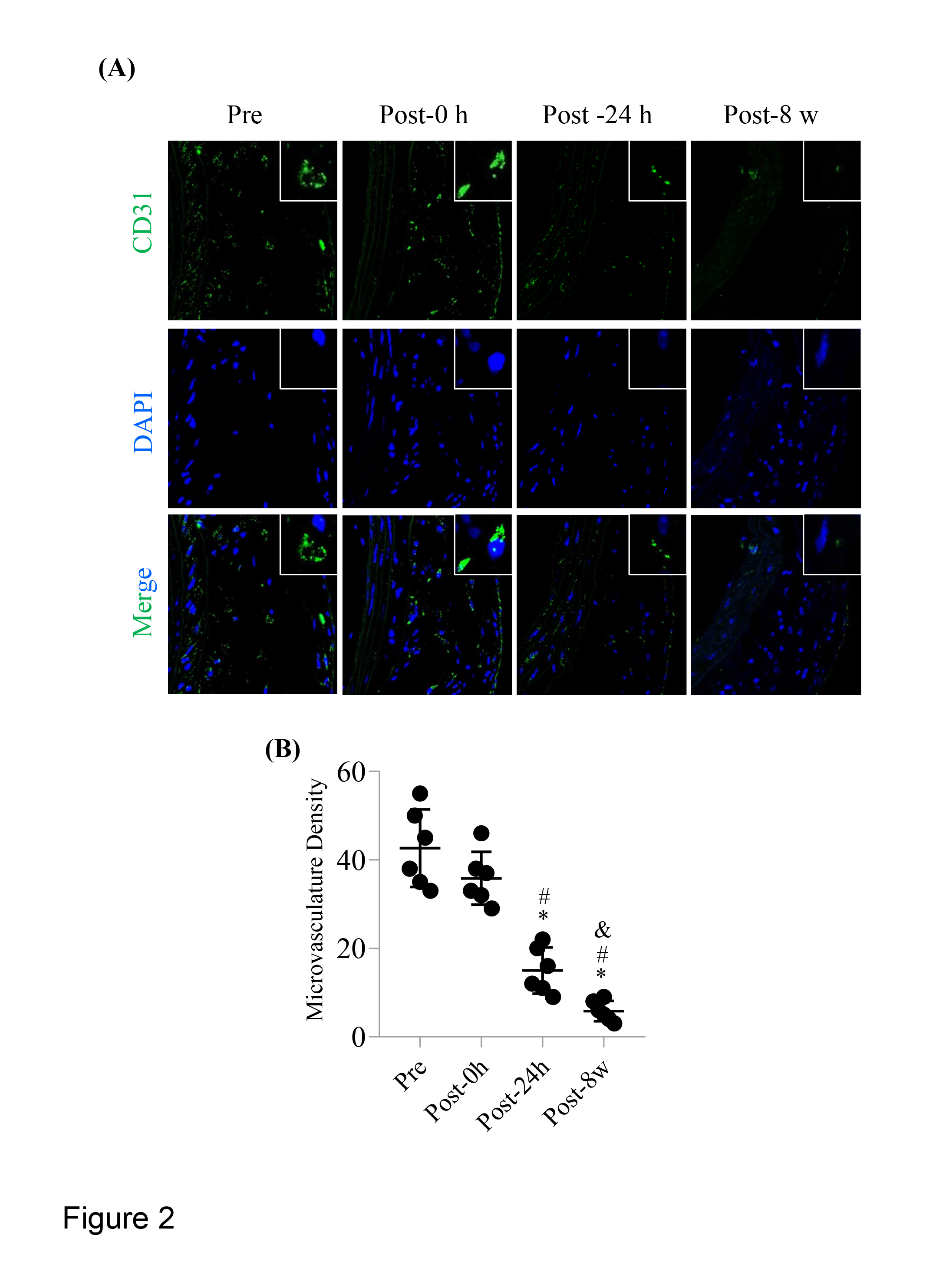

Supplement: Supplementary file 2 [file Image_2.JPEG]

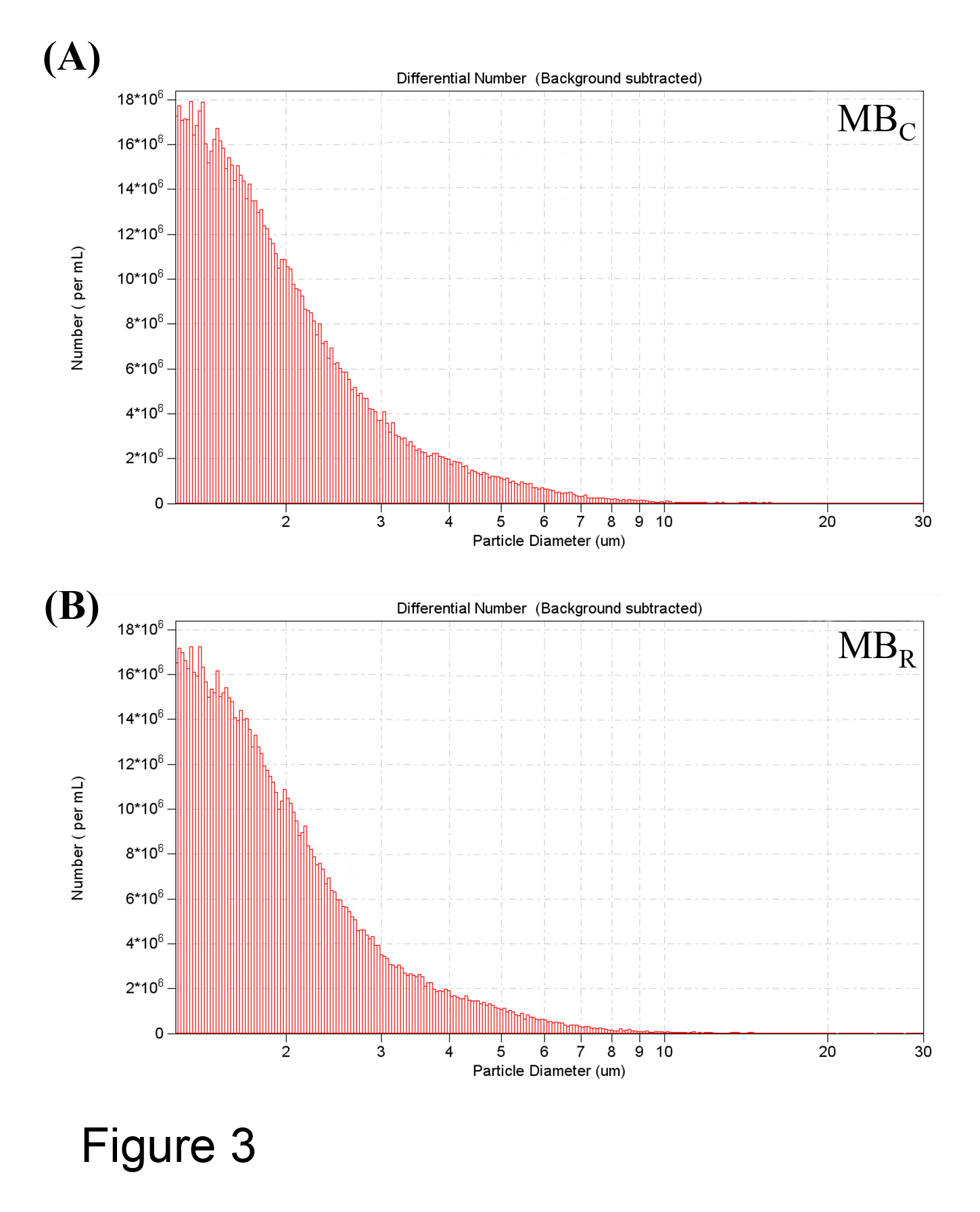

Supplement: Supplementary file 3 [file Image_3.JPEG]
