# Supplementary material for: Effects of diagnostic ultrasound with cRGD-microbubbles on simultaneous detection and treatment of atherosclerotic plaque in ApoE−/− mice
Source: Front Cardiovasc Med. 2022 Jul 22;9:946557. doi: 10.3389/fcvm.2022.946557 (PMC9354833; doi:10.3389/fcvm.2022.946557)
Supplement: Supplementary file 4 [file Presentation_1.PDF]

Supplementary Material

Figure legends

Supplementary Figure 1 The red blood cells reduces decline at time after $DUMB_R$ treatment.

(A) Representative images of immunolabeling for TER-119 before treatment and at 24 h, 8 w, and 12 w after treatment with $DUMB_R$ at an MI of 1.5. (B) The average number of red blood cells was quantified. Data represent the mean ± standard deviation. n=6 per group. *$P<0.05$ vs. Pretreatment, #$P<0.05$ vs. Post-24 h.

Supplementary Figure 2 $DUMB_R$ treatment reduces microvasculature density in plaques.

(A) Representative images of immunofluorescence staining for CD31 before treatment and at 0 h, 24 h, and 8 w after treatment with $DUMB_R$ at an MI of 1.5. (B) Quantification of microvasculature density in plaques. n=6 per group. *$P<0.05$ vs. pretreatment; #$P<0.05$ vs. Post-0 h; & $P<0.05$ vs. Post-24 h.

Supplementary Figure 3 The particle size distribution of the two types of MBs.

The particle size distribution of the MB_C (A) and MB_R (B) was characterized with a Coulter counter.
