# Supplementary material for: Effects of diagnostic ultrasound with cRGD-microbubbles on simultaneous detection and treatment of atherosclerotic plaque in ApoE−/− mice
Source: Front Cardiovasc Med. 2022 Jul 22;9:946557. doi: 10.3389/fcvm.2022.946557 (PMC9354833; doi:10.3389/fcvm.2022.946557)
Supplement: Supplementary file 5 [file Table_1.DOCX]

**Supplementary Table 1** The mean diameter and concentration of MB_C_ and MB_R_ respectively (x ± s, n=6).

|  | MB_C_ | MB_R_ |
| --- | --- | --- |
| Mean diameter (μm) | 2.55±0.31 | 2.54±0.32 |
| Concentration (×10^9^ microbubbles/mL) | 1.15±0.18 | 1.16±0.16 |
